# Understanding the drivers of the public value of e-government: Validation of a public value e-government adoption model

**DOI:** 10.3389/fpsyg.2022.962615

**Published:** 2022-09-13

**Authors:** Isaac Kofi Mensah, Guohua Zeng, Deborah Simon Mwakapesa

**Affiliations:** ^1^School of Business Administration, Fujian Jiangxia University, Fuzhou, China; ^2^School of Economics and Management, Jiangxi University of Science and Technology, Ganzhou, China; ^3^School of Civil and Surveying Engineering, Jiangxi University of Science and Technology, Ganzhou, China

**Keywords:** e-government, e-government services, public value, behavioral adoption, public value e-government adoption model

## Abstract

This study examined the factors driving the public value of e-government from the viewpoint of the Chinese people. The usage of ICT through e-government systems must generate the adequate corresponding public value that can motivate the acceptance of e-government services. The sample 517 data generated from Chinese citizens were analyzed using AMOS 23 software by undertaking the structural equation model system of analysis. The results show that constructs such as information quality, service parameters, user orientation, efficiency, openness, and responsiveness were significantly related to the public value of e-government. Additionally, the research validated that the public value of e-government has a direct influence on the behavioral intention to adopt e-government services. The managerial and practical implications of these research findings on the public value of e-government and the acceptance of e-government services are dissected meticulously.

## Introduction

Public value has emerged as a new dimension in e-government. E-government is the application of information technologies to provide higher standards of innovation in the administration of government operations and systems (Capistrano, 2020; Mouna et al., 2020). E-government activities should have the capacity to enhance the comprehensive and totality of government and public sector performance (Savoldelli, 2013; Twizeyimana and Andersson, 2019). Public value is considered as the value produced by the government via services, laws, and regulations, and can be a vital factor in determining the performance of government programs and activities such as e-government programs (Savoldelli, 2013; Faulkner and Kaufman, 2018; Criado and Gil-Garcia, 2019). It can be used broadly to measure results, the means utilized to provide them, in addition to confidence and lawfulness, and tackles matters like ethos, parity, and responsibility (Savoldelli, 2013; Bojang, 2020). The deep relationship between ideals of public value and e-government has been vividly discussed by scholars (Bouaziz, 2020; Chohan et al., 2020).

The application of ICT in the governance and public administration processes is a vital constituent of the construction of public value within the confines of e-government (Savoldelli, 2013; Bojang, 2021). It is emphasized that e-government systems based on high-tech and managerial modernization could be measured ultimately by examining the likelihood of an upsurge of the public value obtained from the usage of a specific service offered (Savoldelli, 2013; de Sá Medeiros and Forte, 2021). Some have indicated that the creation of public value for people/citizens via services hinges on quality standards such as obtainability of service, satisfaction levels, prominence, issues of fairness, and cost (Cordella and Bonina, 2012; Karkin and Janssen, 2014). Since e-government is based on the provision of citizen-centered programs and actions of government, the measurement and assessment of the public value of e-government concerning the public value generated should also be citizen-centered (Picazo-Vela et al., 2018; Chohan et al., 2020; MacLean and Titah, 2021). E-government is projected as means to determine and increase the public value created by public management systems which indirectly means that e-government strategies can better be measured as per their potential to intensify the public governance capacity of generating public value (Savoldelli, 2013; Picazo-Vela et al., 2015; Lindgren and van Veenstra, 2018).

The goal of the paper is to examine the determinants of the public value of e-government from the understanding of Chinese citizens *via* the validation of a unique Public Value E-Government Adoption Model (PVEGAM). It has been stated that public value-based e-government systems can be examined by looking at the value that people/citizens believe or perceive to get as they consume services delivered by such systems (Luna-Reyes et al., 2016; Roy, 2019; Bouaziz, 2020). The comprehension of the public value of e-government from the citizens' perspective can be instrumental for both policymakers and government sectors since it can empower them to design and deliver e-government systems that provide better public value for every citizen. Through the creation of public value, public institutions meet the aspirations and needs of the people in terms of the service benefits and basic values of an enhanced government. Based on public value dimensions like the quality of information, service delivery parameters, user orientation, efficiency, openness, and responsiveness, this study proposed and validated a public value e-government adoption model (PVEGAM). The proposed and validated PVEGAM is to provide contextualization and specificity of public value within the domain of e-government services adoption. The concept of public value which is the nature of value created by governments *via* services, regulations, and other programs is deemed to be imperative in providing strong support for evaluating the performance of public administration systems and consequently, e-government systems/projects (Savoldelli, 2013; Chohan et al., 2020). Hence the proposed PVEGAM is to provide a framework that contributes to an effective performance evaluation of e-government projects to deliver e-government outcomes that will drive the adoption of e-government services. While previous studies have discussed the concept of public value in the context of e-government (Valle-Cruz, 2019; Bouaziz, 2020; MacLean and Titah, 2021; Scupola and Mergel, 2022), these studies and other bodies of literature however failed to examine the determinants of public value in the domain of e-government and how the public value of e-government can drive the uptake of e-government services. Consequently, this paper fills the conceptual gap and addresses the need for a specific public value of the e-government adoption model to drive the theoretical and practical underpinnings of e-government development and diffusion. The novelty of this study *via* the proposed PVEGAM has demonstrated that the antecedent of the public value of e-government includes factors such as quality of information, service delivery parameters, user orientation, efficiency, openness, and responsiveness. Also, the public value of e-government showed a direct positive effect on the behavioral adoption of e-government services. The following research questions will be interrogated to help achieve the goal of this study: 1) what are the factors driving the public value of e-government? 2) To what magnitude do these factors influence the public value of e-government? 3) To what extent does the public value of e-government affect the adoption intention of e-government services?

The remainder of the article is structured as outlined: literature review, research hypothesis formation, and model, methodology, results and data analysis, discussion of findings with implications, conclusion, and future study.

## Literature review

### E-government

The employment of information and communication technologies (ICT) by the government to promote the efficient delivery of services to businesses and the general public is termed e-government (Andersen and Henriksen, 2006; Almarabeh, 2011; Guo, 2011). The major aim of e-government is to enable the provision of services virtually and to make the life of citizens more comfortable and better (Dwivedi et al., 2012; Ramli, 2017). The concept of e-government has been occasioned due to the difficulties inherent in the traditional delivery of government services, and thus its modernization through ICT is the far better option for government. E-government benefits include easy use of public services, higher accessibility, inclusivity and participation, privacy, and confidentiality to its stakeholders (Rowley, 2011; Meiyanti et al., 2018). For instance, e-government, through the provision of detailed tourist attractions, facilities, and map information for easy location of tourist destinations, can be used to promote tourism (Dewi et al., 2022). Equally, e-government can be instrumental in the design of urban-based smart governance systems to drive the developmental goals of local governments (Hardi and Gohwong, 2020).

Contingent on the manner of the services provided, e-government services may be grouped into Government-to-Citizens (G2C), Citizens-to-Government (C2G), Government-to-Business (G2B), Business-to-Government (B2G), Government-to-Employee (G2E), and Government-to-Government (G2G). The G2C/C2G e-government services ensure the provision of online services and enhance the exchange of information between people and government and vice-visa (Guo, 2011; Meiyanti et al., 2018). On other hand, the G2B/B2G nature of e-government seeks to promote electronic transactions and interactions between business entities and government bodies in terms of e-procurement, the electronic market for government business, and the execution of tenders virtually (Guo, 2011; Meiyanti et al., 2018). Also, the G2E forms of e-government are to transform the internal managerial and communication interaction to create and implement a paperless system (e-office) while the G2G e-government seeks to promote better interaction and information sharing among government offices from the district, municipal, regional, and national levels of government administration system (Guo, 2011; Meiyanti et al., 2018). All these dimensions/forms of e-government illustrate the broader role of government as it attempts to meet the various stakeholders' expectations of public service delivery and reforms needed within government administration systems.

**Figure 1:**
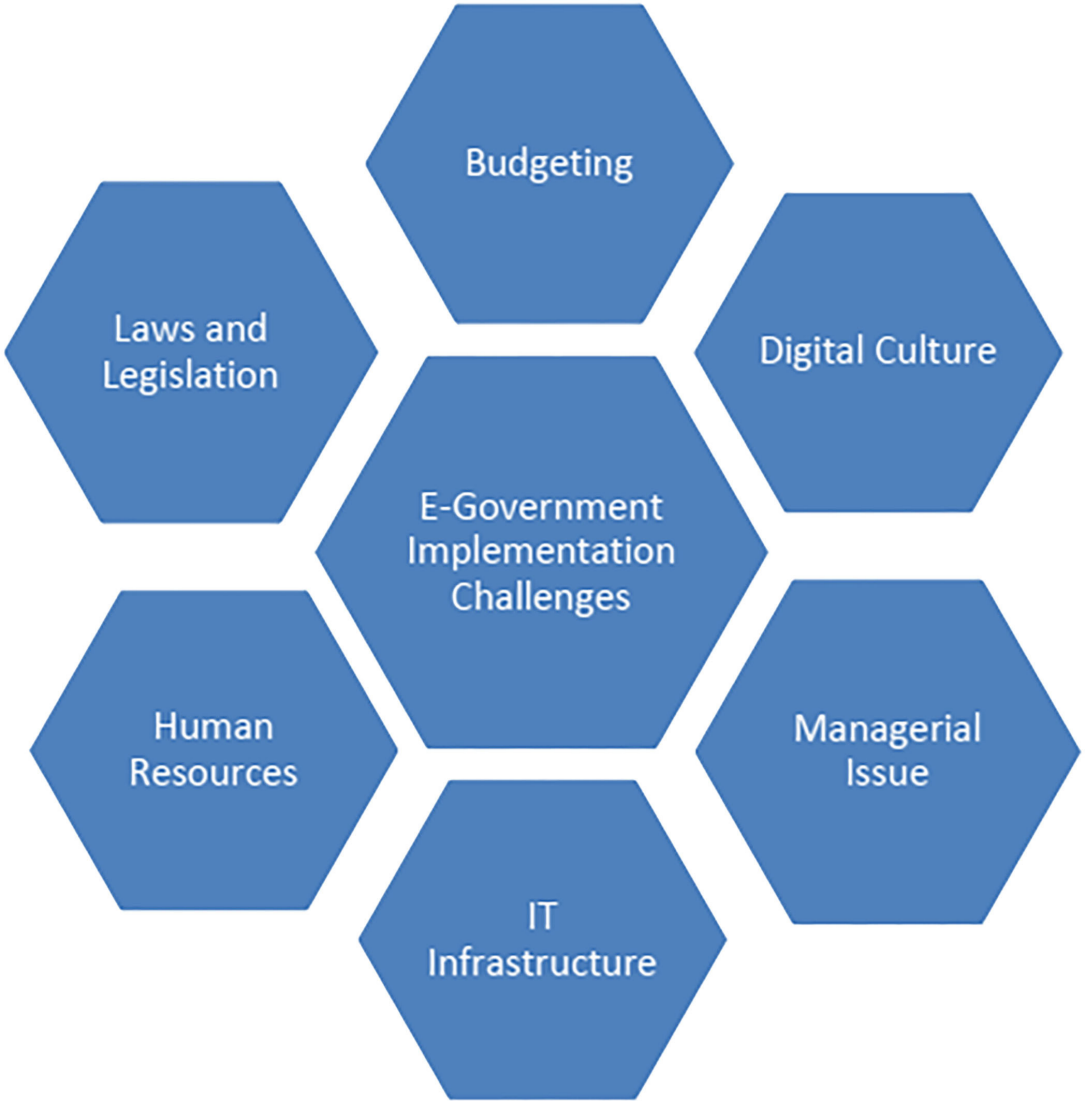
Challenges of e-government implementation.

Despite the advantages connected with the execution of e-government, there are however some major challenges that government and policymakers face in the operation of e-government. These include finance and budgeting, digital culture, managerial issues, IT/ICT infrastructure, human resources/skills, policy regulations, and laws (Figure 1) (Wang and Hou, 2010; Abdelsalam et al., 2012; Abdullah et al., 2013; Meiyanti et al., 2018).

### Public value of e-government

Public value is described as the expectations relating to the delivery of government public services from stakeholders such as citizens, policymakers, civil servants, and tax-payers (Moore, 1995; Castelnovo, 2013). It is vital since, in the use of technology, the value to be obtained from its utilization will influence its adoption (Bannister and Connolly, 2014; Sufna and Fernando, 2020). It has been elaborated that to comprehend the value of e-government, a corresponding comprehension of how the public sector/government entities work is vital (Twizeyimana and Andersson, 2019; Schiff et al., 2021). This is because although both private and public sector organizations offer public services to the people, the modes and underlying concepts/principles vary (Twizeyimana and Andersson, 2019; Brown, 2021). The private sector entities provide services to enhance their profit margins but in the domain of the public sector core principle, government or public entities provide services without necessarily increasing or making a profit (Twizeyimana and Andersson, 2019). Conversely, public sector institutions are rather guided by the major principle of “public value” (Twizeyimana and Andersson, 2019) that is generated by their constituents through the services they offer. A government's key duty is to add value to the people and society at large and this is done through services, laws, regulations, and dedicated policy actions of government (Hui and Hayllar, 2010; Pardo et al., 2021) such as e-government.

Also, the generation of public value should be the primary objective of government institutions since it is through the value that is created that government institutions will fulfill the aspirations of its stakeholders (Jørgensen and Bozeman, 2007; Harrison et al., 2012). The notion of public value is considered as a transformative tool to handle/solve the dynamic social-political influence of information technology on the public/government institutions (Cordella and Bonina, 2012). It has been emphasized that the effect of e-government on public administration effectiveness can best be demonstrated through the public value generated for citizens and the general public (Castelnovo and Simonetta, 2008).

Pubic value has three dimensions. The first dimension has to do with the public value generated through the provision of the high-quality standard of public services and it is driven by issues such as availability, satisfaction, perceived relevance of services, and cost (Kearns, 2004). The second dimension of public value concerns obtaining a certain quality of life outcomes such as better health care, poverty reduction, and reduced environmental pollution (Kearns, 2004). The last element of public value concerns the critical concept of trust in government and its public sector organizations and this concept of trust is vital in influencing the general public to participate and agree to the actions of the government (Kearns, 2004). When the concept of public value is applied to e-government, it provides the following basic criteria from which e-government-specific public value can be understood: the delivery of services that are utilized broadly, higher levels of satisfaction of services provided, improved information, superior accessibility to users, provision of relevant services, cost reduction in accessing services, improved delivery outcomes and enhanced trust interactions between citizens and government entities (Kearns, 2004).

**Figure 2:**
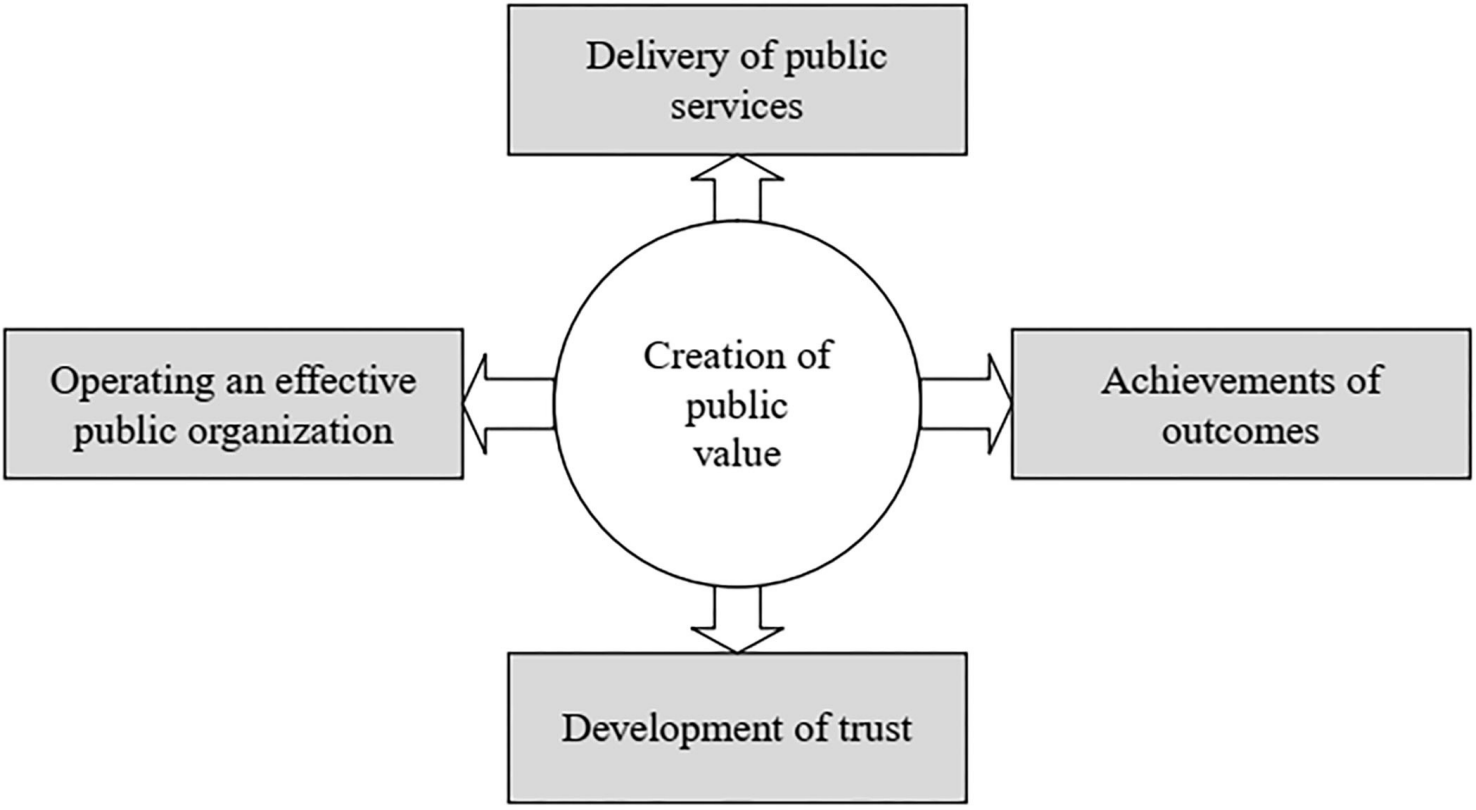
Sources of e-government public value creation (Karunasena et al., 2011).

Furthermore, Karunasena et al. (2011) identified four sources of public value generation *via* e-government and these are supply of services, accomplishment of goals, expansion of confidence, and operating an effective public sector organization. These four foundations of e-government public value construction are depicted in Figure 2.

Apart from these four sources of value creation, other studies have proposed six dimensions of public value of e-government which are inclusive of some of the factors identified by Karunasena et al. (2011). These six dimensions are enhanced government services, better-quality organizational effectiveness, open government capabilities, enriched virtuous behavior and competence, enhanced confidence in management (government), and developed social value and welfare (Twizeyimana and Andersson, 2019). The dimensions of the public value of e-government which include the four sources (Figure 2) of the public value of e-government are described in Table 1.

**Table 1:** Characteristics of the public value of e-government and their descriptions (Twizeyimana and Andersson, 2019).

**Dimensions of public value**	**Descriptions**
1. Improve public services	• Delivery of services to people • Augmented quality and quantity of public services • Offering inclusive public services • Provision of citizen-centered services • Empowered transparency, participation, and collaboration in the delivery of public services
2. Improved administrative efficiency	• Enhanced management of public resources and economy • Decrease in cost and administration burden • Condensed bottleneck and queues in service delivery to citizens • More responsive government system • The robust government in terms of systematic operations, efficiency, effectiveness, flexible and lean) • Facilitated public empowerment and capacity building
3. Open government capabilities	• More open government and increased transparency of public institutions • Improved communication and collaboration programs in the public sector • Enhanced public control and influence on government actions and policies • Improved frequency and intensity of direct participation in decision making • Enhanced political innovations and engagement
4. Improved ethical behavior and professionalism	• Upkeep of fundamental beliefs and constitutional rights • Judicious use of public funds • Facilitation of democratic will • Demand for good information for decisions • Creation of durable and competent institutional capacity
5. Value-added trust and confidence in public institutions (government)	• Ensuring adequate public information and privacy of citizens • Healthier management of public institutions, economy, and resources • Increased transparency and involvement • People have more control of the government's actions and decisions. • Adequate access to government services
6. Improve the social value and wellbeing	• Enhanced social wellbeing • Improved social status, relationship, opportunities, and improved capacity building and empowerment • Influence on individual and household health, security, and satisfaction • Enabled freedom and equal rights • Attainment of superior results in capacities such as peace, security, poverty reduction, public health, high employment, low crime rates, and better educational attainment

## Research hypothesis

### Quality of information

The quality of information generated on e-government systems is critical to determining the value that the public attaches to e-government. Information quality can be understood through people's beliefs concerning the value of information availability (Karunasena and Deng, 2012; Rachmawati, 2019). The main features of quality of information are precision, accuracy, timeliness, and relevance (Wangpipatwong et al., 2005; Gorla et al., 2010). Information accuracy has to do with the veracity of the information provided on e-government sites while timeliness is the degree to which the services (information) delivered are current and updated (Wangpipatwong et al., 2005). The relevance of information is the extent to which the information delivered meets the expectations of the users while information precision indicates that the provided information is presented in a manner that is easy to read and comprehend (Wangpipatwong et al., 2005; Papadomichelaki and Mentzas, 2009; Janita and Miranda, 2018). The quality of the information provided *via* the e-government system must be detailed and comprehensive to adequately meet the service expectations of citizens (Gorla et al., 2010; Karunasena and Deng, 2012). E-government services that achieve these four dimensions of information quality will ultimately impact the public value of e-government. Past scholarships have indicated that the quality of information has a direct impact on the public value of e-government and behavioral adoption (Elenezi et al., 2017; Nulhusna et al., 2017; Almaiah and Nasereddin, 2020). H1 was hence projected.

**H1:** Quality of information is positively associated with the public value of e-government.

### Service delivery parameters

E-government development and implementation are instrumental in ensuring the effectiveness of public service delivery parameters (Karunasena and Deng, 2012). The public value of e-government can be better measured by people's sensitivities regarding the value of the two-way interactions (communications) that empower full real-time interaction between users and government (Karunasena and Deng, 2012; Chohan et al., 2020). These include transactional e-government facilities like the payment of government/public services online, completing and submitting application forms virtually, and searching for archival information and data (Torres et al., 2005a,b; Filgueiras et al., 2019). The abundantly functional use of transactional e-government that can improve the public delivery parameters can drive the public value of e-government. The service delivery parameters have been determined to influence positively the public value of e-government (Karunasena and Deng, 2012). Consequently, H2 was proposed.

**H2:** Service delivery parameter is positively associated with the public value of e-government.

### User-orientation

User orientation relates to a citizen-centered approach and development of e-government services (Karunasena and Deng, 2012; Liang et al., 2019) that meets the aspirations and desires of the general public. The adoption of a citizen-centered attitude to e-government development can ensure the effective delivery of e-government services (Chang et al., 2009; Yotawut, 2018). The user orientation dimensions could include issues such as the development of user-friendly e-government features and the presentation of information that is concise, comprehensive, and detailed (Papadomichelaki and Mentzas, 2009). E-government that conforms to the expected user characteristics and needs will enhance its public value. The direct influence of user orientation on the public value of e-government has been statistically proven by previous studies (Karunasena and Deng, 2012). Accordingly, H3 was proposed.

**H3:** User orientation is positively associated with the public value of e-government.

### Efficiency

One of the crucial features that influences the implementation of e-government is the ability to enhance efficiency not only for government service provision but also for government sector organizations. For instance, the efficiency in e-government can be understood through organizational efficiency, responsiveness, accountability, and openness (Karunasena and Deng, 2010). Organizational efficiency, as a result of e-government, can lead to reducing costs and better utilization of public sector resources that optimizes ICT infrastructure use, re-structuring of government functions, and well-trained public sector staff (Karunasena and Deng, 2012; Nabafu and Maiga, 2012). E-government development to provide higher efficiency will influence the citizen's public value of e-government. In recent literature, it has been revealed that efficiency has a direct significant impact on the public value of e-government (Karunasena and Deng, 2012; Deng et al., 2018b). Consequently, H4 was projected.

**H4:** Efficiency is positively associated with the public value of e-government.

### Openness

The lack of transparency in government dealings is a fundamental reason for introducing e-government as a tool to make government more open and accountable. The concept of transparency in public organizations is desirable, fostered, and empowered by the emergence of e-government and computing technologies (Bannister and Connolly, 2011). Openness has to do with transparency and accountability of services, especially in terms of making decisions, processes, and procedures as well as public information (budgets and expenditure) readily available to citizens (Pina et al., 2010; Milić et al., 2018). According to Karunasena and Deng (2010), these are key indicators of openness. Perception of openness and transparency in the provision of public services powered by e-government will have an uninterrupted effect on the public value of e-government. Studies have illustrated that openness has a positive impact on the public value of e-government (Karunasena and Deng, 2012). Consequently, H5 was proposed.

**H5:** Openness is positively associated with the public value of e-government.

### Responsiveness

Responsiveness is the degree to which government sector organizations adhere to the requests and demands of citizens (Ngonzi and Sewchurran, 2019). Responsiveness in e-government is the expectation that citizens have regarding timely responses to their demands/inquiries through the e-government system (Karunasena and Deng, 2012; Milosavljević et al., 2017). Government agencies can meet the requirement of swift response by stating clearly the minimum of days required for a particular service to be provided or processed and thus achieving a more responsive public administration system (Karunasena and Deng, 2012; Eom et al., 2018). The higher degree of responsiveness in the provision of government services via e-government will surely influence people's perception of the public value of e-government. It has been demonstrated that responsiveness is directly related to the public value of e-government (Karunasena and Deng, 2012). Consequently, H5 was proposed.

**H6:** Responsiveness is positively associated with the public value of e-government.

### Public value

Public value is considered as the total value created/generated to meet the expectations of diverse stakeholders i.e., citizens and businesses by the government (Kelly et al., 2002; Karunasena and Deng, 2012). In terms of e-government, the public value is the use of e-government systems to improve the provision of government services that meet the demands of citizens and the general public (Dolan, 2015). Citizens are considered the core of the public value idea which evaluates the summation of benefits arising from government policies and actions toward citizens (Alford and O'Flynn, 2008; Meynhardt, 2009; Karunasena and Deng, 2012). Value creation should be the cardinal principle that drives the operations of public sector organizations in the implementation of government policies and programs such as e-government (Meynhardt, 2009). It is expected that the higher the public value created through e-government in terms of open data, data privacy, and ant-corruption measures (Valle-Cruz, 2019), the higher the adoption of e-government services would be. Research has indicated that public value created through e-government has a direct significant impact on citizens adopting e-government and mobile government services (Li and Shang, 2020; Wang et al., 2020). H7 was therefore proposed.

**H7:** Public value of e-government is positively associated with the intention to adopt e-government services.

## Research model

**Figure 3:**
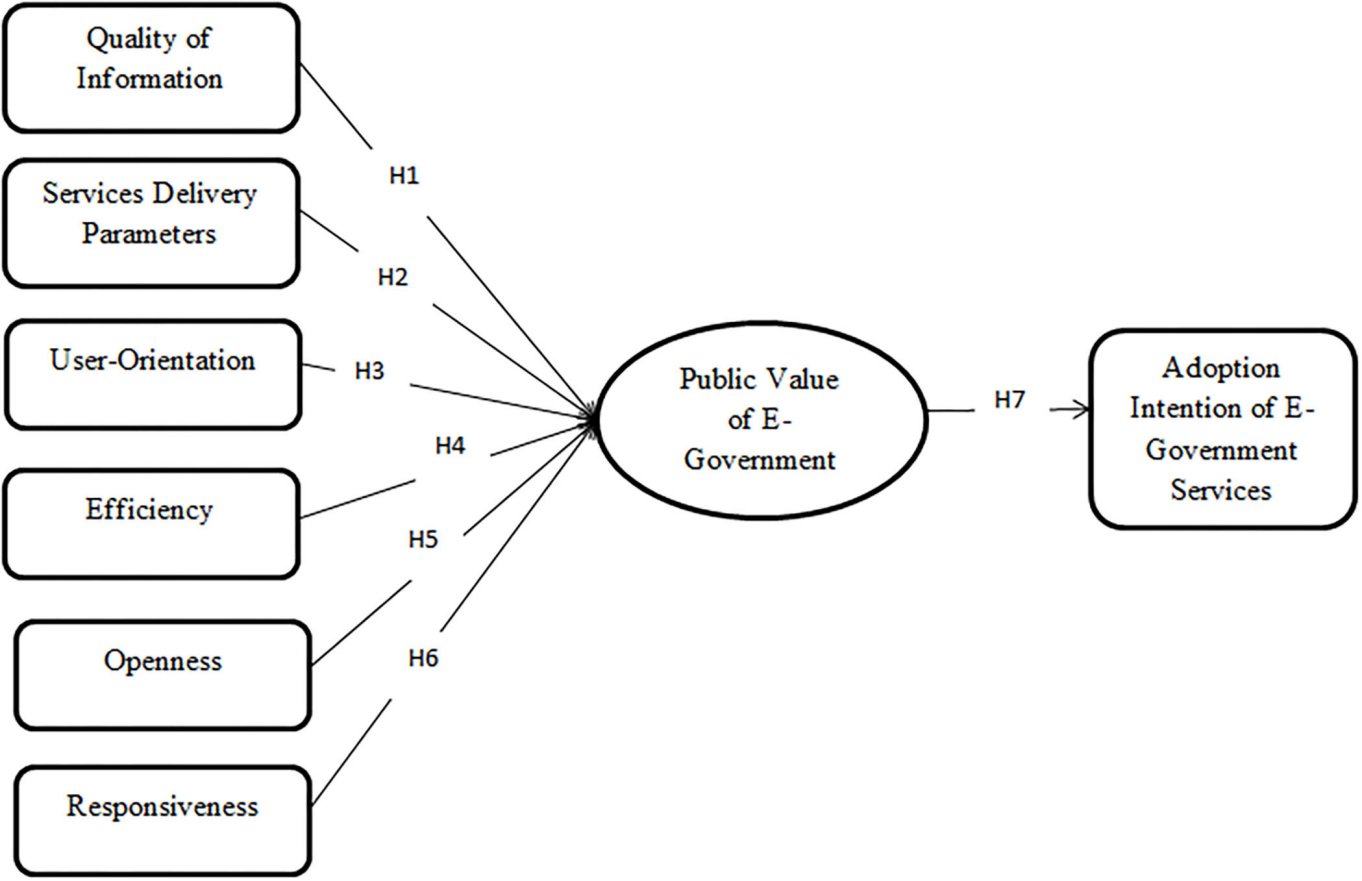
Proposed public value of e-government adoption model (PVEGAM).

The proposed research model based on the research hypotheses developed is shown in Figure 3.

## Research methodology

To adequately test the proposed public value of the e-government adoption model (PVEGAM), a thorough review of existing literature was undertaken to identify the constructs to be included in the model. The proposed model contained eight carefully selected variables adapted from the following sources: information quality (Wang and Liao, 2008; Karunasena and Deng, 2012; Osman et al., 2014), service delivery parameters (Karunasena and Deng, 2012), user-orientation (Kim et al., 2009; Karunasena and Deng, 2012; Osman et al., 2014), efficiency (Kolsaker and Lee-Kelley, 2008; Pathak et al., 2012), openness (La Porte et al., 2002; Jaeger and Bertot, 2010; Karunasena and Deng, 2012), responsiveness (Gauld et al., 2010; Ayyash et al., 2011; Karunasena and Deng, 2012), public value (Deng et al., 2018b; Liang et al., 2019), and behavioral adoption (Davis, 1989; Venkatesh et al., 2003). The research items are attached as Appendix A.

These constructed variables constituted the research questionnaire that was administered to a cross-section of Chinese citizens in the city of Ganzhou, specifically within the university community of Jiangxi University of Science and Technology. The variables were measured on Likert five-point scale which varied from strongly disagree (1) to strongly agree (5). The questionnaire was pretested and piloted before it was finally administered. Pre-testing and piloting are essential to ensure that the content of the questionnaire has clarity and is clearly understood by the respondents. Though the pre-testing and piloting were instrumental in restructuring the content of the questionnaire, the results of the pre-testing and piloting were not incorporated into the final data analysis.

The questionnaire was hosted online from September to October 2020 and the link was shared on social media platforms (individual and group WeChat) for the respondents to complete. The social media platform was selected because it is the most used social interaction application among the Chinese. It was also because of the convenience it provided to reach respondents with ease and for faster collection of research data. A total of 517 valid responses was received. These valid responses were then captured and used for the data analysis. The structural equation modeling technique was used to analyze the data generated with the help of AMOS 23 software and SPSS.

### Common method bias

Scholars have indicated that the presence of common method bias (CMB) has the potential to cause a problem when researching especially when a single source is used to acquire research data (Podsakoff et al., 2003); therefore, this study examined the possibility of common method bias in our research. The common method bias is considered a serious challenge since it is perceived as the main source of measurement errors and has the potential to affect the validity of the conclusion concerning the interaction between measures/constructs (Podsakoff et al., 2003; Min et al., 2016). Harman's single-factor test was used to check the existence of CMB through the use of exploratory factor analysis. This was done to see if a single factor will emerge or account for the majority of the covariances between the measures. If this is not the case, then it is evident that CMB does not exist (Chang et al., 2020). As per the single factor analysis conducted, no single factor accounted for more than 37% of the variance of the majority measures, which was below the threshold of 50% (Tehseen et al., 2017). Accordingly, it can be said that issue of CMB does not exist in our study.

## Data analysis

### Profile of participants

The information of the study sample is shown in Table 2. There were more female (58%) respondents as compared to males (42%), the majority of the respondents were between the ages of 31–40 (40.4%), and most of them were master's degree holders (43.9%).

**Table 2:** Profile of participants.

**Item**	**Description**	**Frequency**	**Percentage**
Gender	Male	217	42
	Female	300	58
Age	18–26	95	18.4
	26–30	120	23.2
	31–40	209	40.4
	41–50	51	9.9
	50+	42	8.1
Education	Undergraduate Degree	144	27.9
	Masters	227	43.9
	Ph.D.	94	18.2
	Others	52	10.1

### Measurement model

The results of the measurement model are shown in Table 3. The measurement dimensions were examined by the computing factor loadings, Cronbach's alpha, composite reliability, and the average variance extracted (AVE). Cronbach's alpha and composite reliability are acceptable when they have values above 0.70 (Henseler et al., 2016). The factor loadings and average variance extracted were recommended to be above 0.70 and 0.50 respectively (Henseler et al., 2009; Hair et al., 2010) for items to be considered valid. As indicated in Table 3, all the quality dimensions for good and acceptable measurement model outputs have been achieved. This thus meets the convergent validity of the items used in this study. A second level assessment was undertaken to determine the discriminant validity of the items used by using the square roots of AVE and the cross-loading matrix. The square roots of AVE are recommended to be greater than its correlation with variables for discriminant validity to exist. Additionally, the diagonal variables should have higher values as compared to the items in its related columns and rows if discriminant validity is to be achieved. As indicated in Table 4, the conditions for discriminant validity to occur have been met and thus confirm the discriminant validity of the items used in the study.

**Table 3:** Measurement model.

**Constructs**	**Code**	**Loadings**	**Cronbach's**	**Composite**	**AVE**
			**alpha**	**reliability (CR)**	
Information quality	QI1	0.995	0.957	0.959	0.885
	QI2	0.935			
	QI3	0.890			
Service delivery parameters	SDP1	0.926	0.945	0.949	0.862
	SDP2	0.811			
	SDP3	0.936			
User-orientation	UO1	0.971	0.971	0.971	0.719
	UO2	0.925			
	UO3	0.979			
Efficiency	EF1	0.915	0.932	0.936	0.832
	EF2	0.808			
	EF3	0.901			
Openness	OP1	0.990	0.959	0.960	0.890
	OP2	0.878			
	OP3	0.959			
Responsiveness	RESP1	0.895	0.968	0.969	0.811
	RESP2	0.971			
	RESP3	0.995			
Public value	PV1	0.995	0.969	0.970	0.814
	PV2	0.900			
	PV3	0.971			
Adoption intention	AI1	0.982	0.975	0.975	0.829
	AI2	0.935			
	AI3	0.974			

**Table 4:** Discriminate validity.

	**AI**	**EF**	**OP**	**PV**	**QI**	**RESP**	**SDP**	**UO**
AI	**0.912**							
EF	0.843	**0.839**						
OP	0.565	0.572	**0.900**					
PV	0.660	0.784	0.868	**0.930**				
QI	0.782	0.669	0.772	0.672	**0.960**			
RESP	0.568	0.471	0.577	0.662	0.881	**0.969**		
SDP	0.772	0.581	0.678	0.776	0.685	0.886	**0.949**	
UO	0.673	0.464	0.377	0.657	0.783	0.883	0.786	**0.973**

Bold indicates (diagonal) square root of AVE. AI, Adoption Intention; EF, Efficiency; OP, Openness; PV, Public value; IQ, Information Quality; RESP, Responsiveness; SDP, Service Delivery Parameters; UO, User-Orientation.

### Structural model

**Figure 4:**
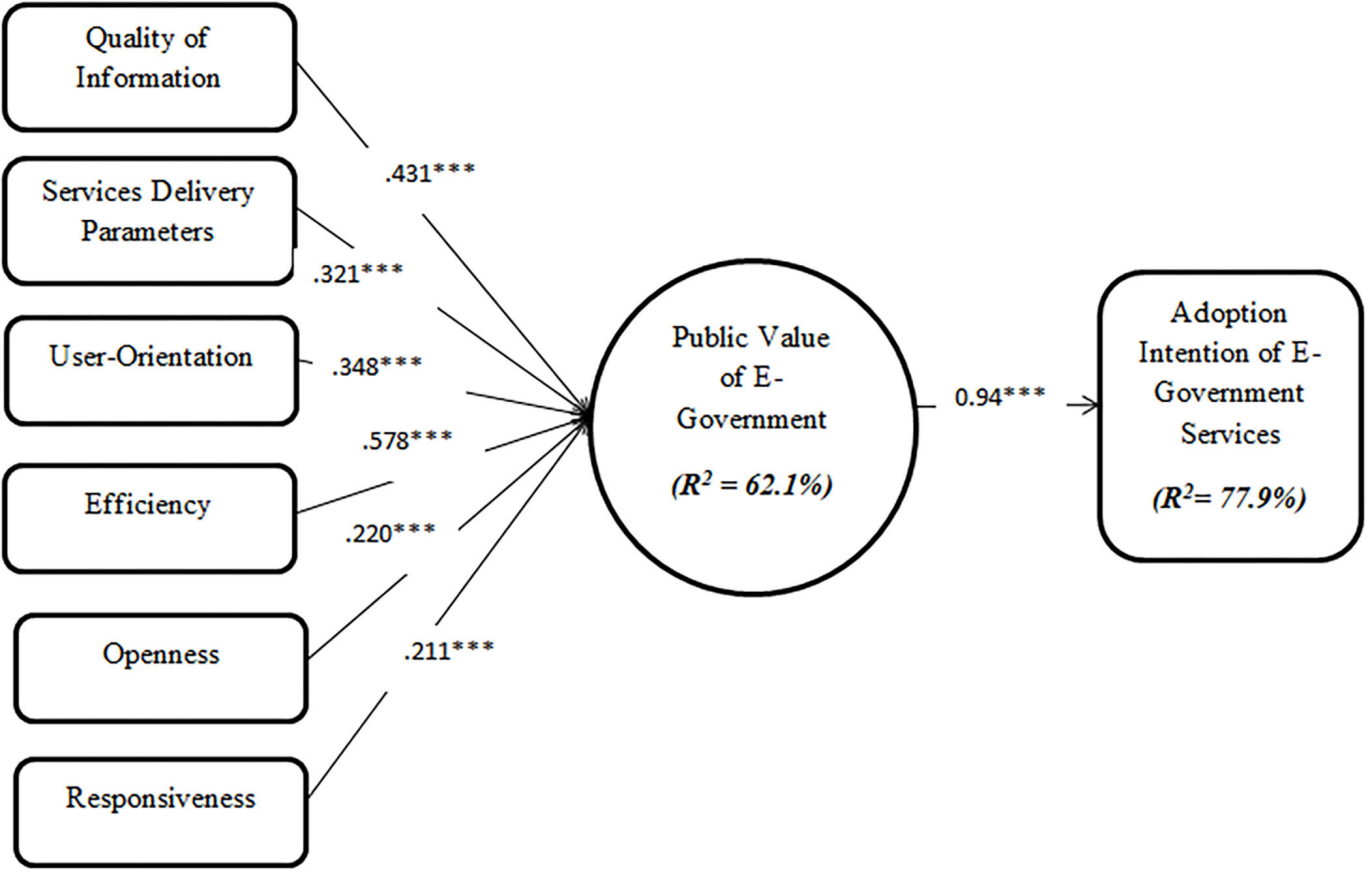
Validated research model.

The results of the research hypotheses tested are indicated in Table 5. It was revealed that quality of information (β = 0.431, *p* < 0.05) and service delivery parameters (β = 0.321, *p* < 0.05) were significant predictors of the public value of e-government. Hence H1 and H2 were supported. Also, user-orientation (β = 0.348, *p* < 0.05) and efficiency (β = 0.578, *p* < 0.05) were found to be significant determinants of the public value of e-government. H3 and H4 were thus supported. Furthermore, openness (β = 0.220, *p* < 0.05) and responsiveness (β = 0.211, *p* < 0.05) were also found to be significant predictors of public value of e-government. Accordingly, H5 and H6 were supported. Finally, the public value of e-government was determined to be a significant determinant of the behavioral intention to adopt e-government services (β = 0.960, *p* < 0.05). The graphical depiction of the validated research model is illustrated in Figure 4.

**Table 5:** Results of hypothesis.

**Hypotheses**	**Path**	**β**	**Std. Error**	**T-value**	**Sign**.	**Supported**
H1	QI→ PV	0.431	0.042	10.168	^***^	Yes
H2	SDP→ PV	0.321	0.053	6.122	^***^	Yes
H3	UO→ PV	0.348	0.045	7.680	^***^	Yes
H4	EF→ PV	0.578	0.040	14.434	^***^	Yes
H5	OP→ PV	0.220	0.041	5.366	^***^	Yes
H6	RESP→ PV	0.211	0.040	5.246	^***^	Yes
H7	PV→ AI	0.960	0.002	406.505	^***^	Yes

AI, Adoption Intention; EF, Efficiency; OP, Openness; PV, Public value; IQ, Information Quality; RESP, Responsiveness; SDP, Service Delivery Parameters; UO, User-Orientation.

*** p < 0.05.

## Discussion

The public value of e-government has attracted great attention in recent years but there is a limited amount of literature in this regard, especially in the context of China. This study thus was determined to examine the factors influencing the public value of e-government from the Chinese citizen's perspective and its ultimate impact on the adoption of e-government services. The analyses of the results have vividly demonstrated that the entire proposed hypotheses were supported. Specifically, constructs such as quality of information, service delivery parameters, user orientation, efficiency, openness, and responsiveness were significant in influencing the Chinese citizens' public value of e-government. In addition, the public value of e-government, in turn, was found to be directly related to the behavioral acceptance of e-government services.

The direct significant influence of quality of information and service delivery parameters on the public value of e-government demonstrates the important role these indicators play in the public value creation of e-government. The quality of information of e-government services such as accuracy, timeliness, relevance, and precision will enhance the citizens' comprehension of the public value of e-government. The findings are in line with other studies that have demonstrated that the quality of information (Deng et al., 2018a; Wang and Teo, 2020) and service delivery parameters (Karunasena and Deng, 2012) are directly related to the public value of e-government. Also, it supports studies that showed that information and service equality drives the public value dimension of e-government (Alhanatleh et al., 2022).

In addition, this paper has revealed that constructs such as user orientation and efficiency have a direct and significant impact on the public value of e-government services. This means that better public value can be created through the designing of public services that are user-oriented and meet the efficiency expectations of the people. The ability of users to easily download forms, pay online, contact government officials, access simple and searchable government websites, avail single point of services, have concise website addresses, and equipped with frequently asked questions will enhance the user-orientation perspective and thus will ultimately influence the public value of e-government. In terms of the efficiency dimension, e-government structures that re-design local and central government systems and processes, have IT empowered contact points, and retrain local government officers and public servants with ICT and e-government skills will determine the public value of e-government. This finding is in line with findings of previous studies on the direct impact of user orientation on the public value of e-government (Karunasena and Deng, 2012; Deng et al., 2018a).

Furthermore, this study demonstrated that openness and responsiveness of e-government systems have a significant influence in determining the public value of e-government. One of the cardinal principles that prompted the introduction of e-government in the public administration systems is the lack of openness and responsiveness from the local and central government agencies in the administration of government business. Well-designed e-government systems that can ensure transparency through acts such as putting government policy and programs online for consultation and input, the release of public organizations' budgets and expenditures virtually, declaration of tenders online, empowering people to engage and make compliant virtually and the making available of official government and public servants contact/information online holds well for the public value of e-government. This is emphasized by Valle-Cruz (2019) who found that public value can be created through anti-corruption, open data, access to information, and privacy measures. On the other hand, well-structured responsive e-government systems influence increase in public value of e-government through acts such as automatic feedback to submissions made online, making of inquiries online, feedback to inquiries done through e-government, and the tracking and tracing of applications submitted online. The result of the direct effect of openness and responsiveness on the public value of e-government is corroborated by past research that has indicated that these two constructs are instrumental in driving the public value of the e-government (Karunasena and Deng, 2012; Deng et al., 2018a; Roy, 2019; Chohan et al., 2020).

Finally, the study validated that the public value of e-government has a direct impact on the behavioral adoption of e-government services. This means that ICT through e-government systems should be used to generate greater public value in e-government to better meet the service dimension and expectations of people. An e-government system that is well designed and executed to enhance the administrative and interaction potential of government systems in terms of the provision of quality information, transparency and accountability, openness, responsiveness, organizational efficiency, and orientated toward the user will drive better comprehension of the public value of e-government which will in turn influence the use of e-government services. The public value of e-government dimensions such as improved public services, managerial effectiveness, open government, ethical conduct and competence, better trust in government, and enhance the social value and welfare of citizens as indicated by Twizeyimana and Andersson (2019) and Abdulkareem and Ramli (2021) can be instrumental in driving the adoption of e-government services. The significant impact of the public value of e-government on the behavioral intention to use e-government services is supported by previous research that reported similar findings that the nature of public value generated in e-government can determine the level of acceptance of e-government services (Mellouli et al., 2020).

### Theoretical implications

This study has contributed to e-government and public value creation in e-government literature by demonstrating the drivers of public value and acceptance of e-government services within the context of Chinese society through validated PVEGAM. The constructs examined like the quality of information, service delivery parameters, user orientation, efficiency, openness, and responsiveness accounted for 62.1% of the factors influencing the public value of e-government from the perspective of the sample population. In addition, the public value of e-government, in turn, accounted for 77.9% of the elements driving the behavioral acceptance of e-government services.

### Practical implication

The first implication of the results of this study is the validated direct influence of quality of information and service parameters on the public value of e-government. The creation of public value through e-government cannot be achieved without the designing of e-government services that are integrated with information quality dimensions. That is the quality of information delivered through e-government should meet certain criteria of relevancy, currency, interpretability, believability and reputation, accuracy, authoritative, and objectivity for it to create and generate the needed public value expectations of e-government. Policymakers in terms of e-government development should be concerned about the development of swift, accurate, pertinent, and precise/concise information and services if the public value of e-government is to be attained.

Another key result of this study is the significant impact of user orientation and efficiency on the public value of e-government which implies the development and diffusion of e-government. Technology-related applications often are concerned with how users can successfully interact with such applications or services. This thus calls for developers and designers of e-government projects to develop e-government systems that will yield better user orientation features such as easy to download and upload, easy navigation and browsing, accessibility, and ease of use. Also, efficiency implies the use of e-government systems that will re-design and re-align systems and processes to better achieve organizational goals and service parameters. Efficiency through e-government should help decrease the budget for processing and administration of public sector agencies; enhance better connectivity between citizens and public organizations, ensure adequate public information sharing, and result in shorter interaction among agencies.

Furthermore, this research confirmed that openness and responsiveness have a direct effect on the public value of e-government. The transparency of government agencies, sector ministries, and departments, by making all information and relevant programs readily available and open to citizens and the general public, can generate public value in e-government. It thus calls on the government to be open and demonstrate high levels of accountability and transparency to citizens through an effective e-government system. Exhibiting and achieving demonstrable openness and transparency in public administration and governance has the prospective to improve the citizens' perspective of the public value of e-government. Demonstrable openness and transparency can be achieved through the government's proactive dissemination of information, provision of needed documents and materials, public engagement or meetings, and leaks from whistleblowers which can reduce the level of corruption within and outside government.

The responsiveness dimension impact on the public value of e-government does indicate the need to redesign government processes and systems to be highly responsive to the service requirements of the citizens. It is only in this way that citizens can understand the public value of e-government programs. Policymakers should therefore design responsive e-government that will respond timely to emails and feedback, tracing of applications and status online, etc. The failure to achieve a responsive e-government system tends to disrupt the smooth interaction between citizens and government which may lead to the citizens not harboring the desire to connect or contact the government either physically, *via* phone, or by post.

Additionally, the public value of e-government was found to have a significant impact on the intention of citizens to adopt e-government services. This study provides empirical evidence for policymakers and practitioners of e-government to focus on creating greater public value in e-government since it has a subsequent effect on the decision of users to take up e-government services. For a government to be successful in e-government services, much attention must be paid to developing e-government systems that will create better public value for citizens. Government and agencies in charge of e-government must improve perceptions of citizens regarding the public service provided through e-government within the context of public value generated while delivering public services. These public values in e-government can be created through the provision of e-government systems that provide quality information, is user-orientated, ensure efficiency, is responsive to the needs and service desire of users, and ultimately is environmentally sustainable. Public value in the e-government architecture should be utilized to create improved administrative efficiency, improved public services, and improved social values. Once these public value dimensions of e-government can be noticed by users/citizens it will motivate them to use e-government services.

## Conclusion

Having demonstrated the importance of public value in accessing public services through e-government and its subsequent effect on the uptake of e-government services, this study set out to determine the factors influencing the public value of e-government from the perspective of Chinese citizens. According to the proposed hypotheses, research framework, and the data analysis conducted using Smart PLS structural equation modeling technique, this study has statistically supported all the hypothesized interactions in this study. Particularly, this study has shown that the determinants of the public value of e-government are quality of information, service delivery parameters, user orientation, efficiency, openness, and responsiveness. In addition, the public value of e-government was revealed to influence the adoption intention of e-government services. These findings in addition to their research implications can provide policymakers and government agencies with the pathway to improve the public value of e-government and its subsequent effect on the higher uptake of e-government services.

## Research limitation and future work

The drivers of the public value of e-government may not be the same across the board and thus the constructs and model validated in this study may not yield the same results if applied in different country contexts. Also, the sample use may not be wholly representative which implies that the interpretation of the results should not be over-generalized. Additionally, not all the factors influence the public value of e-government; therefore, future studies should seek to integrate other factors such as trust in government, cost of internet bandwidth, and quality of service. Lastly, future work should add moderating and mediating constructs to the current research model to fully comprehend the public value of e-government.

## Data availability statement

The original contributions presented in the study are included in the article/supplementary material, further inquiries can be directed to the corresponding author/s.

## Ethics statement

Ethical review and approval was not required for the study on human participants in accordance with the local legislation and institutional requirements. Written informed consent for participation was not required for this study in accordance with the national legislation and the institutional requirements.

## Author contributions

IM: conceptualization, methodology, data collection, analysis, and writing–original draft preparation. GZ: writing and literature review, revisions, and secure funding. DM: literature review, revisions, and editing. All authors contributed to the article and approved the submitted version.

## Funding

This research was supported and funded by the Youth Jinggang Scholars Program in Jiangxi Province (QNJG2020047) and National Social Science Fund of China (Project numbers: 18BGL246 and 21XGL021).

## Conflict of interest

The authors declare that the research was conducted in the absence of any commercial or financial relationships that could be construed as a potential conflict of interest.

## Publisher's note

All claims expressed in this article are solely those of the authors and do not necessarily represent those of their affiliated organizations, or those of the publisher, the editors and the reviewers. Any product that may be evaluated in this article, or claim that may be made by its manufacturer, is not guaranteed or endorsed by the publisher.

## Appendix

### Appendix A: Research Items

**Quality of Information (QI)**

QI1: accurate information

QI2: Information that is updated

QI3: Clear and comprehensible information

**Service Delivery Parameters (SDP)**

SDP1: Ability to access services online

SDP2: fill and submit forms online and downloads forms

SDP3: Ability to pay online

**User-orientation (UO)**

UO1: e-government site contains useful links

UO2: e-government sites have frequently asked questions (FAQs)

UO3: e-government website is simple and friendly

**Efficiency (E)**

E1: Improve ICT infrastructure

E2: IT-supported public service counters

E3: re-structured government offices/ministries

**Openness (OP)**

OP1: Public policy documents online

OP2: Availability of budget and expenditure online

OP3: making tenders online

**Responsiveness (RESP)**

RESP1: Make enquires online

RESP2: Tracking of complaints and cases online

RESP3: Timely feedback to forms submitted online

**Public Value (PV)**

PV1: e-government can enhance information sharing and collaboration

PV2: e-government can improve administrative innovation

PV3: e-government ensure transformation in government service delivery

**Adoption Intention (AI)**

AI1: I plan to use e-government service

AI2: I will recommend e-government services to others

AI3: I will use e-government in the future
